# Overexpression of OsAGO18 Promotes Early Seedling Development and Root Elongation in Rice

**DOI:** 10.3390/plants15101580

**Published:** 2026-05-21

**Authors:** Cheng Tang, Xiaoliang Shan, Xinwei Liao, Qiwang Hu, Xiaoxiao Hu, Ran Wei, Hongwei Zhao

**Affiliations:** 1 State Key Laboratory of Agricultural and Forestry Biosecurity, College of Plant Protection, Nanjing Agricultural University, Nanjing 211800, China; 2021202038@stu.njau.edu.cn (C.T.); liaoxinwei@stu.njau.edu.cn (X.L.); 2024802299@stu.njau.edu.cn (Q.H.); 2023102056@stu.njau.edu.cn (X.H.); 2024102061@stu.njau.edu.cn (R.W.); 2 Liaoning Institute of Economic Forestry, Liaoning Academy of Agricultural Sciences, Dalian 116031, China; sdausxl@126.com

**Keywords:** *OsAGO18*, IAA, *OsGRF6*, growth, development

## Abstract

Argonaute (AGO) proteins are central components of RNA silencing. While OsAGO18 is a known defense factor in antiviral immunity, its involvement in basal development and its transcriptomic behavior during fungal stress remains to be fully elucidated. In this study, based on its specific dual-localization in chloroplasts and processing bodies (P-bodies), we investigated the pleiotropic effects of OsAGO18 through transcriptomic network analysis of rice responding to the blast fungus *Magnaporthe oryzae* B.C. Couch. Our analysis revealed that the OsAGO18-mediated co-expression network is highly correlated with ribosome biogenesis and cell wall organization. Notably, the analyzed datasets reveal that this growth-related network is significantly suppressed upon *M. oryzae* challenge, highlighting a transcriptomic shift in *OsAGO18* during the growth-to-defense transition. Phenotypic evaluations demonstrated that *OsAGO18* overexpression accelerates early seedling growth and primary root elongation by promoting endogenous indole-3-acetic acid (IAA) accumulation, whereas *ago18* mutants maintain basal growth rates without significant IAA fluctuations, reflecting robust genetic compensation within the highly redundant AGO family. Mechanistically, our integrated analysis suggests that OsAGO18 acts as a putative molecular decoy to sequester miR396d, thereby relieving the repression of the Growth-Regulating Factor *OsGRF6* and triggering downstream auxin-dependent cascades. Collectively, our findings highlight OsAGO18 as a pivotal regulator of early seedling development and characterize its transcriptomic responsiveness to biotic stress, providing a plausible molecular link between post-transcriptional RNA regulation and rice growth coordination.

## 1. Introduction

Early seedling establishment and root system architecture are fundamental determinants of plant survival and subsequent crop yield [1]. These developmental processes are heavily driven by the spatiotemporal dynamic distribution of phytohormones, most notably IAA [2,3]. To maintain precise cellular homeostasis and coordinate rapid growth, plants have evolved robust post-transcriptional regulatory networks. Among these, microRNAs (miRNAs) act as master tuners by directing the cleavage or translational repression of target messenger RNAs (mRNAs) involved in hormone biosynthesis and signal transduction [4,5].

The execution of miRNA-directed silencing strictly relies on Argonaute proteins, the core catalytic components of the RNA-induced silencing complex (RISC) [6]. The rice (*Oryza sativa* L.) genome encodes a highly diverse AGO family consisting of 19 members, which exhibit distinct expression patterns and functional specializations [7]. Among them, OsAGO18 is a monocot-specific AGO protein that has garnered significant attention for its critical role in broad-spectrum antiviral immunity [8]. Wu et al. systematically demonstrated that upon viral infection, *OsAGO18* expression is highly induced to competitively sequester host miR168. This “decoy” activity relieves the suppression of *OsAGO1*, thereby boosting the antiviral RNAi machinery [8]. While predominantly categorized as a defense-responsive factor, subsequent genetic evidence indicated that *OsAGO18* is also required for normal plant development. For example, silencing *OsAGO18* alters the accumulation of endogenous miRNAs and phased secondary small RNAs (phasiRNAs), resulting in stunted growth and severe male sterility [9]. While predominantly categorized as a defense-responsive factor under viral stress, its baseline physiological role in vegetative tissues, particularly during the critical stages of early seedling development, remains largely unexplored. Therefore, investigating *OsAGO18* provides a unique opportunity to uncover how a single AGO protein can switch between executing developmental programs under normal conditions and deploying immune responses during stress.

In normal developmental contexts, diverse plant architectures and growth parameters are tightly controlled by specific miRNA-target modules. A well-established paradigm is the miR396-Growth-Regulating Factor (GRF) regulatory network [10]. The miR396 family targets the GRF transcription factors, which are highly conserved regulators of cell proliferation, root elongation, and floral organogenesis [10,11]. Specifically, previous molecular analyses have confirmed that elevated expression of *OsGRF6*, a primary target of miR396d, directly promotes auxin biosynthesis and signaling, leading to enhanced primary root growth and altered inflorescence architecture in rice [11]. Recent molecular profiling further defined this regulatory axis, demonstrating that OsGRF6 acts as a direct transcriptional activator of OsYUCCA1, a key flavin monooxygenase-like enzyme in the tryptophan-dependent indole-3-acetic acid biosynthesis pathway. By binding to the *OsYUCCA1* promoter, *OsGRF6* drives substantial endogenous IAA accumulation to promote secondary branching and morphological growth. Although OsGRF6 concurrently targets OsWRKY82 to modulate jasmonic acid (JA) signaling for pathogen defense, its developmental output is primarily governed by the IAA signaling cascade [12].

In this study, we aimed to uncover the developmental functions of *OsAGO18* in rice. We first analyzed its three-dimensional structural features and subcellular localization, revealing a specific dual-localization pattern in chloroplasts and P-bodies. By integrating transcriptome-wide co-expression networks (WGCNAs) with phenotypic evaluations of transgenic lines, we demonstrated that *OsAGO18* is a positive regulator of early seedling growth and auxin-dependent root elongation under normal physiological conditions. Furthermore, molecular complex modeling combined with published RNA immunoprecipitation data suggested that OsAGO18 acts as a decoy to sequester miR396d, thereby protecting the OsGRF6 transcript from degradation. Collectively, our cytological, physiological, and transcriptomic data delineate a post-transcriptional regulatory axis in which *OsAGO18* coordinates basal rice development and auxin homeostasis prior to its recruitment for immune responses.

## 2. Results

### 2.1. OsAGO18 Specifically Localizes to Chloroplasts and Processing Bodies

**Figure 1:**
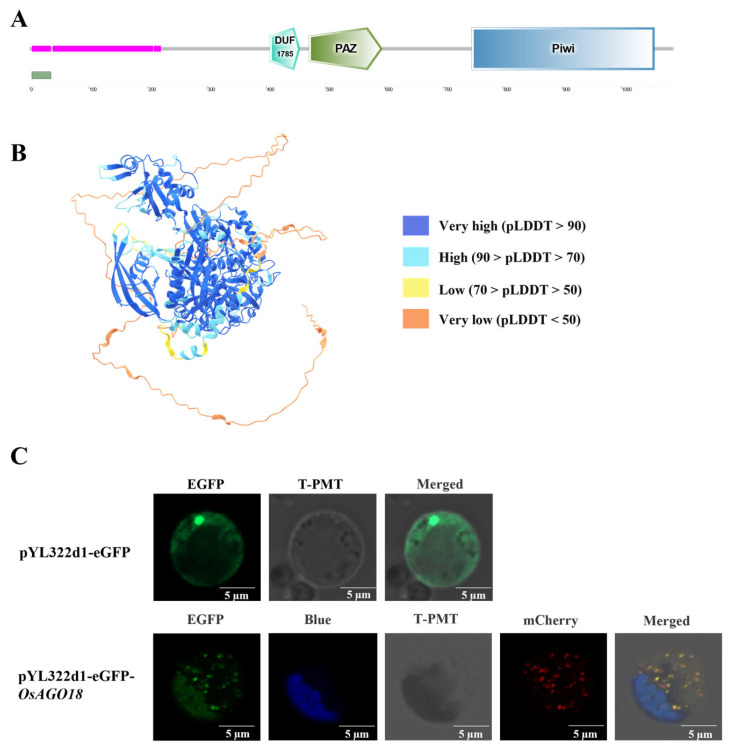
Structural features and subcellular localization patterns of OsAGO18. (**A**) Schematic representation of the conserved domain architecture of OsAGO18, highlighting the DUF1785, PAZ, and Piwi domains. (**B**) The 3D protein structure of OsAGO18 predicted by AlphaFold. The color gradient from blue to orange represents the predicted local distance difference test (pLDDT) confidence scores. (**C**) Subcellular localization of OsAGO18 in rice protoplasts. The empty vector (pYL322d1-eGFP) was used as a negative control (top panel). The bottom panel displays the co-localization of eGFP-OsAGO18 (green) with the P-body marker pUC19/CF-mChe (red) and chloroplast autofluorescence (blue). To clearly distinguish the dual-localization signals, the natural red autofluorescence of chloroplasts was pseudocolored blue. T-PMT represents the bright-field images. Scale bars, 5 μm.

To gain insights into the molecular characteristics of OsAGO18, we first analyzed its conserved domains and predicted three-dimensional (3D) structure. Domain architecture analysis revealed that OsAGO18 possesses typical AGO family characteristics, sequentially containing a DUF1785 domain, a central PAZ domain, and a C-terminal Piwi domain (Figure 1A). Based on this structural framework, a 3D model of the OsAGO18 protein was generated utilizing AlphaFold (Figure 1B). The core regions of the protein exhibited very high structural reliability, with predicted local distance difference test (pLDDT) scores greater than 90.

To elucidate the spatial distribution of OsAGO18 in vivo, we conducted subcellular localization assays using rice protoplasts. A transient expression vector carrying the OsAGO18 coding sequence fused with enhanced green fluorescent protein (eGFP) was constructed. As shown in Figure 1C (top panel), the fluorescence signal of the empty vector control (pYL322d1-eGFP) was ubiquitously distributed throughout the cytoplasm and nucleus. In contrast, the eGFP-OsAGO18 fusion protein exhibited a specific localization pattern. To determine its exact cellular compartments, we co-expressed eGFP-OsAGO18 with the processing body (P-body) marker pUC19/CF-mChe [13]. As shown in Figure 1C (bottom panel), the green fluorescent puncta of OsAGO18 merged with the red fluorescent signals of the P-body marker. Simultaneously, a significant portion of the eGFP-OsAGO18 signal co-localized with chloroplast autofluorescence (which was pseudocolored blue to clearly distinguish it from the red P-body signals). Collectively, the dual accumulation of OsAGO18 in chloroplasts (the hub of metabolism) and P-bodies (the site of RNA regulation) provides a cytological basis for its potential role in coordinating complex cellular programs.

### 2.2. OsAGO18 Is Highly Conserved Among Oryza Species and Monocotyledonous Plants

**Figure 2:**
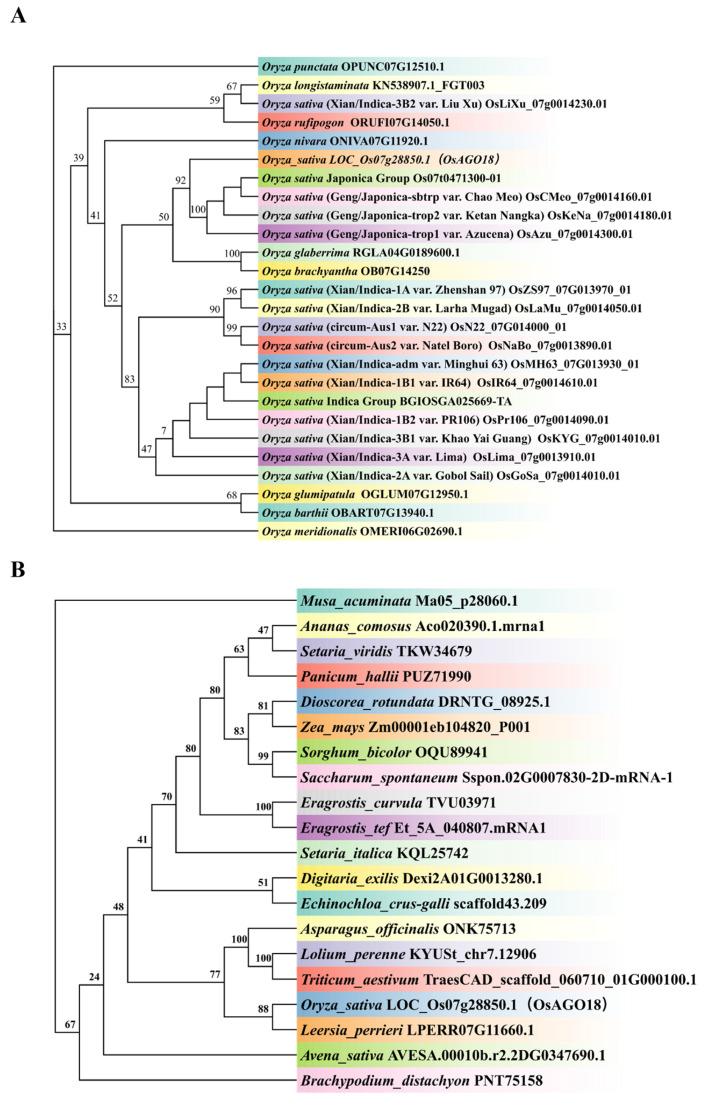
Phylogenetic analysis of OsAGO18 and its homologs. (**A**) A neighbor-joining (NJ) phylogenetic tree of OsAGO18 homologs within the *Oryza genus*, encompassing various cultivated subspecies (*japonica* and *indica*) and wild rice relatives. (**B**) A phylogenetic tree showing the evolutionary relationships of OsAGO18 orthologs across representative monocotyledonous species. The numbers at the branch nodes indicate bootstrap values. *OsAGO18* (*O. sativa* LOC_Os07g28850.1) is highlighted in the trees.

Given the specific dual-localization of OsAGO18, we next asked whether this functional constraint is evolutionarily conserved. To trace the evolutionary history and sequence conservation of OsAGO18, we performed a phylogenetic analysis. We retrieved homologous protein sequences of OsAGO18 and constructed phylogenetic trees. Within the *Oryza genus*, the phylogenetic analysis revealed that OsAGO18 is highly conserved (Figure 2A). It clusters tightly with orthologs from various cultivated rice varieties, including both japonica and indica subspecies, as well as wild rice species such as *Oryza rufipogon* and *Oryza nivara*.

To further evaluate the evolutionary relationship of AGO18 across a broader taxonomic range, we expanded the analysis to typical monocotyledonous plants. As illustrated in Figure 2B, OsAGO18 groups distinctly within the *Poaceae* (grass) family clade, exhibiting close genetic distances with homologs from major agricultural crops, including wheat (*Triticum aestivum*), maize (*Zea mays*), and sorghum (*Sorghum bicolor*). These phylogenetic results suggest that AGO18 is an evolutionarily conserved protein among monocots, implying a fundamental and potentially conserved biological function across these species.

### 2.3. Transcriptomic Analysis Reveals OsAGO18 as a Basal Growth Regulator with Stress-Responsive Network Dynamics

Based on its specific dual-localization in chloroplasts and P-bodies (Figure 1C), combined with its established function in antiviral defense [8], we reasoned that *OsAGO18* might exert pleiotropic effects on both fungal immunity and basal developmental processes. To address this, we leveraged 34 high-resolution transcriptome datasets tracking the responses of diverse rice genotypes—including the wild-type Nipponbare (NPB), the susceptible line LTH, and the resistant line IRBLkm-Ts—to the blast fungus *Magnaporthe oryzae* B.C. Couch (strain Guy11). While these datasets capture the molecular dynamics of biotic stress, our analysis here primarily focuses on elucidating the role of OsAGO18 in driving baseline developmental programs and cellular homeostasis.

**Figure 3:**
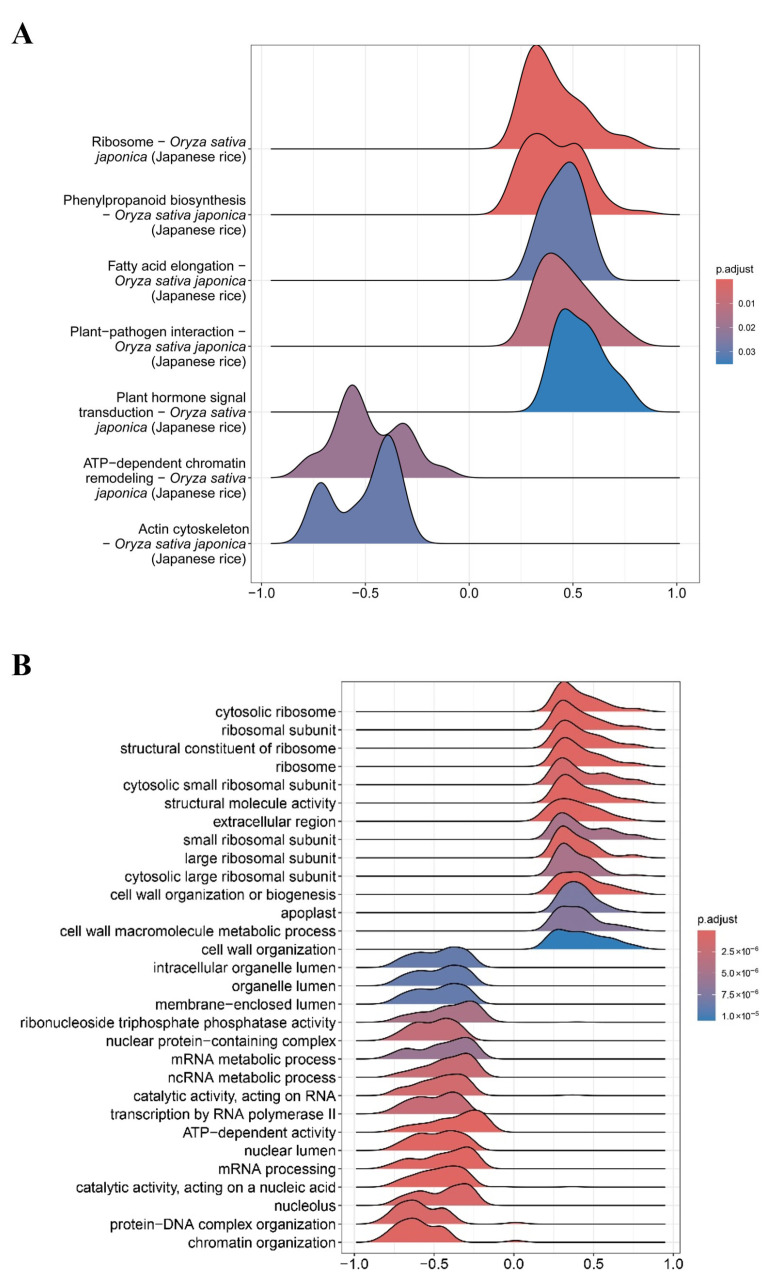
Single-gene enrichment analysis uncovers the biological pathways co-expressed with *OsAGO18*. The analysis was performed using 34 publicly available transcriptome datasets to identify global expression correlations. (**A**) Ridge plot of KEGG pathway enrichment analysis for genes correlated with *OsAGO18*. Peaks shifted to the right (*p*-values) indicate pathways positively correlated with *OsAGO18*, while peaks to the left indicate negative correlations. (**B**) Ridge plot of GO enrichment analysis highlighting the top functional terms associated with *OsAGO18*. The color gradient represents the adjusted *p*-values.

Kyoto Encyclopedia of Genes and Genomes (KEGG) pathway analysis revealed that genes positively correlated with *OsAGO18* expression are predominantly enriched in “Ribosome”, “Phenylpropanoid biosynthesis”, and “Fatty acid elongation” (Figure 3A). Phenylpropanoid metabolism is a critical upstream pathway for lignin biosynthesis and cell wall reinforcement. Consistently, Gene Ontology (GO) enrichment analysis confirmed a strong positive correlation with “structural constituent of ribosome”, “cytosolic ribosome”, and “cell wall organization or biogenesis” (Figure 3B). Rapid de novo protein synthesis and massive cell wall remodeling are the fundamental physiological requirements for seed germination and early seedling growth.

Intriguingly, the GO analysis also revealed that *OsAGO18* is negatively correlated with terms such as “mRNA processing”, “RNA processing”, and “chromatin organization” (Figure 3B). This transcriptional signature perfectly aligns with our subcellular localization findings (Figure 1C). Given that OsAGO18 specifically localizes to P-bodies—cytoplasmic ribonucleoprotein granules dedicated to mRNA turnover, storage, and translational repression—these data suggest a functional link. Collectively, we hypothesize that OsAGO18 operates within P-bodies to dynamically reprogram mRNA metabolism, thereby directing cellular resources toward the robust translation machinery and cell wall construction essential for rice development.

To further dissect the complex co-expression networks involving *OsAGO18*, we subjected the transcriptome datasets to Weighted Gene Co-expression Network Analysis (WGCNA). As shown in Supplementary Figure S1, the module–trait relationship heatmap identified several distinct gene modules highly correlated with specific physiological states. Notably, *OsAGO18* was assigned to the MEyellow module. Strikingly, the MEyellow module exhibited an extremely significant positive correlation with the “normal” physiological baseline condition (r = 0.94, *p* = 6 × 10^−19^). Conversely, this module displayed strong negative correlations with disease-associated traits, including both “resistant” and “sensitive” responses, as well as early post-inoculation time points (e.g., time24). These module-level network dynamics strongly corroborate our transcriptome-wide co-expression analysis. Although previous studies have well-documented the crucial role of *OsAGO18* in plant immunity, our distinct correlation pattern suggests that it also exerts a fundamental function in driving robust early development and baseline cellular homeostasis under normal conditions. This points towards a pleiotropic role for *OsAGO18*, actively coordinating basal growth machinery prior to its recruitment for biotic stress responses.

### 2.4. OsAGO18 Positively Regulates Early Seedling Growth and Root Elongation via IAA Accumulation

To experimentally validate the bioinformatics predictions regarding the role of OsAGO18 in early development, we evaluated the germination and seedling growth phenotypes using various genetic materials. These included the wild-type NPB, two independent overexpression lines (*AGO18-OE-#15* and *AGO18-OE-#20*), and loss-of-function mutant lines (*ago18-T17* and *C-ago18*) (Supplementary Figure S2).

**Figure 4:**
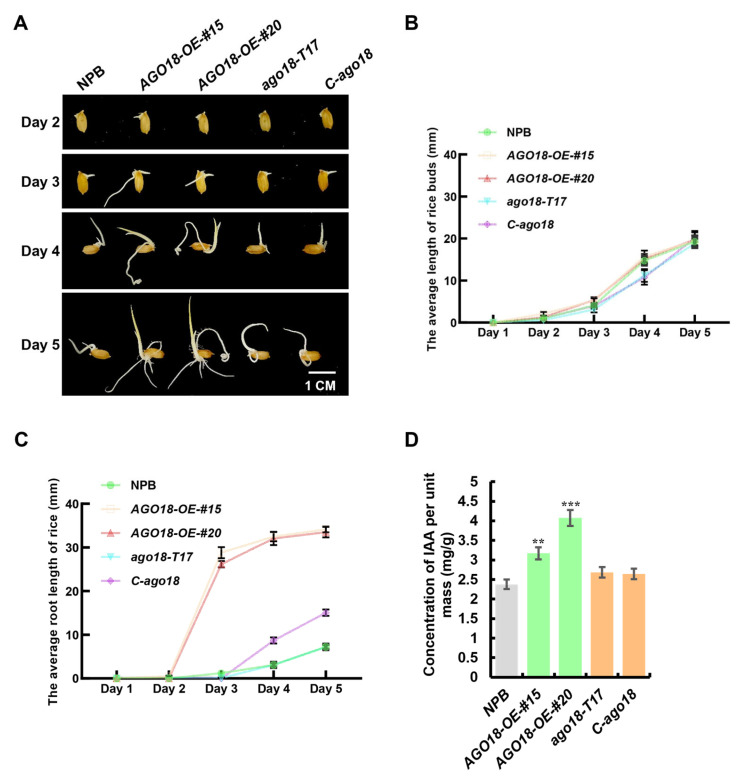
*OsAGO18* promotes early seedling growth and auxin accumulation. (**A**) Representative images of germinating seeds from the wild type (NPB), *OsAGO18* overexpression lines (*AGO18-OE-#15*, *AGO18-OE-#20*), and mutant lines *(ago18-T17*, *C-ago18*) over a 5-day time course. Scale bar = 1 cm. (**B**) Statistical analysis of the average length of rice buds over the 5-day period. (**C**) Statistical analysis of the average root length over the 5-day period. (**D**) Quantification of endogenous IAA concentration per unit mass in the seedlings. Values are represented as mean ± SD. Asterisks indicate significant differences compared to the NPB control according to Student’s *t*-test (** *p* < 0.01, *** *p* < 0.001).

A continuous 5-day observation of the germination process revealed distinct developmental trajectories among the genotypes (Figure 4A). Phenotypic quantification demonstrated that the *AGO18-OE* lines exhibited an accelerated elongation of rice buds compared to the NPB control, whereas both *ago18* mutant lines displayed a basal growth pattern similar to the wild-type NPB (Figure 4B). The most prominent developmental difference was observed in primary root elongation. While both *AGO18-OE* lines displayed a rapid increase in primary root length starting from Day 3, the *C-ago18* mutant lines exhibited a mild growth overshoot, resulting in roots slightly longer than those of the wild-type NPB at Days 4 and 5, though substantially shorter than the *AGO18-OE* lines (Figure 4A,C). These observations indicate that OsAGO18 acts as a positive regulator of early root development, while the mild mutant phenotype suggests functional compensation by other AGO family members.

Given that IAA is the master phytohormone driving cell division and root elongation, we hypothesized that *OsAGO18* might modulate auxin homeostasis to promote growth. To test this, we quantified the endogenous IAA concentration in the seedlings. As depicted in Figure 4D, the IAA content was significantly elevated in both *AGO18-OE-#15* and *AGO18-OE-#20* lines compared to the NPB wild type. In contrast, the IAA levels in the mutant lines remained comparable to the background (Figure 4D). Collectively, these results demonstrate that the massive root elongation in the overexpression lines is driven by enhanced auxin accumulation, whereas the mild growth overshoot in the single knockout mutants occurs independently of IAA spikes, likely reflecting broader genetic redundancy and basal network readjustment.

### 2.5. OsAGO18 Potentially Sequesters miR396d to Derepress the Growth-Regulating Factor OsGRF6

**Figure 5:**
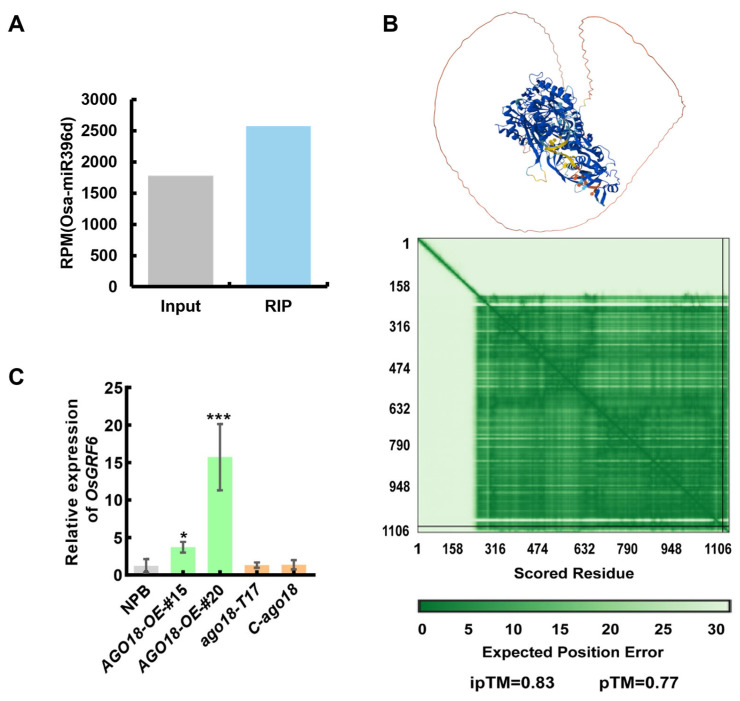
OsAGO18 relieves *OsGRF6* suppression by potentially sequestering miR396d. (**A**) Enrichment of Osa-miR396d in the OsAGO18 RNA immunoprecipitation fraction compared to the total input. Reads per million (RPM) values were directly extracted from the published supplementary deep sequencing dataset [8]. (**B**) In silico prediction of the OsAGO18-miR396d complex utilizing AlphaFold3. The upper panel shows the predicted 3D conformation of the protein-RNA interaction. The lower panel displays the Predicted Aligned Error (PAE) heatmap, along with the interface predicted template modeling (ipTM) and predicted template modeling (pTM) scores, indicating high prediction confidence. (**C**) Relative expression levels of *OsGRF6* in the wild type (NPB), *AGO18-OE* lines, and *ago18* mutant lines determined by qRT-PCR. *18S rRNA* was used as an internal reference for qRT-PCR. Values are represented as mean ± SD. Asterisks indicate significant differences compared to the NPB control according to Student’s *t*-test (* *p* < 0.05, *** *p* < 0.001).

To elucidate the molecular mechanism underlying *OsAGO18*-mediated root elongation and IAA accumulation, we investigated its potential small RNA binding partners. As an AGO protein, OsAGO18 functions primarily by loading specific microRNAs. We mined the previously published OsAGO18 RNA immunoprecipitation (RIP) sequencing dataset [8]. This analysis revealed an enrichment of Osa-miR396d in the OsAGO18 RIP fraction compared to the input (Figure 5A), suggesting a potential in vivo association. To further evaluate the physical interaction between the OsAGO18 protein and the miR396d transcript in silico, we utilized AlphaFold3 to model the protein-RNA complex. The modeling yielded a favorable predicted structural interaction, supported by a low expected position error in the Predicted Aligned Error (PAE) heatmap (Figure 5B). Notably, the complex prediction achieved high interface and overall structural scores (ipTM = 0.83, pTM = 0.77), supporting the feasibility of a stable spatial binding conformation between OsAGO18 and miR396d.

It is well documented that miR396d post-transcriptionally represses *OsGRF6*, a crucial node in auxin signaling and plant architecture. We hypothesized that the sequestration of miR396d by OsAGO18 would relieve the targeted degradation of *OsGRF6*. Consistent with this decoy hypothesis, quantitative real-time PCR (qRT-PCR) assays demonstrated that the transcript levels of *OsGRF6* were significantly elevated in the *AGO18-OE* lines compared to the NPB wild type. Conversely, *OsGRF6* expression remained at basal levels in the loss-of-function mutant lines (Figure 5C). Taken together, these results support a working model wherein OsAGO18 modulates early seedling growth by sequestering miR396d, thereby derepressing *OsGRF6* to trigger downstream auxin-dependent developmental cascades.

To molecularly validate the activation of these auxin signaling cascades, we examined the transcript levels of the early auxin-responsive gene *OsIAA10* and the auxin efflux carrier *OsPIN9*. Given that elevated endogenous auxin rapidly induces the transcription of *Aux/IAA* family genes, these transcripts serve as reliable readouts for active auxin pathways. qRT-PCR analysis showed that both *OsIAA10* and *OsPIN9* were significantly upregulated in the *AGO18-OE* lines compared to the NPB control, while their expression remained at basal levels in the *ago18* mutants (Supplementary Figure S3). These transcriptional changes are consistent with the elevated IAA content and root elongation phenotypes, confirming that *OsAGO18* positively modulates auxin-dependent seedling development.

## 3. Discussion

AGO proteins act as the essential catalytic engines of small RNA-directed silencing pathways [14]. In rice, OsAGO18 has been extensively characterized as a viral-inducible defense factor that confers broad-spectrum antiviral immunity [8]. However, whether OsAGO18 exerts fundamental functions beyond biotic stress responses has remained largely unexplored. In this study, we demonstrate that OsAGO18 is a highly conserved monocot protein that strictly governs early seedling development and basal physiological homeostasis. Our combination of phenotypic analysis, cytological observation, and transcriptomic profiling positions OsAGO18 as a critical positive regulator of auxin-dependent root elongation.

### 3.1. P-Body Localization Highlights a Potential Role in Basal mRNA Reprogramming

A striking observation from our cytological analysis is the specific aggregation of OsAGO18 in P-bodies and chloroplasts. P-bodies are conserved, membrane-less cytoplasmic ribonucleoprotein granules primarily responsible for mRNA turnover, storage, and translational repression [15,16]. This spatial distribution perfectly correlates with our GO enrichment analysis, which revealed a strong negative correlation between OsAGO18 and “mRNA processing” under normal baseline conditions. During early seed germination and seedling establishment, cells must undergo massive transcriptomic reprogramming, switching from dormant storage mRNAs to rapid de novo protein synthesis [17,18,19]. We hypothesize that OsAGO18 operates within P-bodies to transiently sequester specific mRNAs or miRNAs, thereby dynamically modulating the translational machinery to support the rapid cell wall organization and ribosome biogenesis observed in our transcriptomic correlation network. Furthermore, our WGCNA assigned *OsAGO18* to a gene module highly active under normal physiological states rather than post-inoculation states, suggesting that OsAGO18 might participate in coordinating basal growth prior to its recruitment as a defense component. Nevertheless, it is important to note that the current link between the specific subcellular localization of OsAGO18 and the observed downstream developmental phenotypes remains a correlative speculation. Direct causal evidence linking its precise role within P-bodies to specific developmental outputs awaits further experimental validation.

### 3.2. OsAGO18 Acts as a Molecular Decoy to Modulate the miR396d-OsGRF6 Axis

The core mechanistic insight of our study is the proposition of OsAGO18 as a putative molecular decoy for miR396d during early development. Unlike typical AGO proteins that directly slice target mRNAs, OsAGO18 was previously shown to employ a unique competitive binding strategy. Wu et al. elegantly demonstrated that upon viral infection, OsAGO18 sequesters host miR168 and miR528 to relieve the suppression of OsAGO1, thereby boosting antiviral defenses [8]. Our findings remarkably expand this paradigm into the realm of plant developmental biology. By mining the published OsAGO18 RIP dataset [8] and employing precise in silico AlphaFold3 complex modeling, we uncovered supporting evidence for a potential physical association between OsAGO18 and miR396d. While previous analyses of this exact dataset correctly highlighted the robust enrichment of miR168 and miR528 during viral infection, our phenotype-driven data mining uncovered the concurrent binding of miR396d. We certainly do not exclude the presence or functional importance of the miR168 and miR528 modules; however, these defense-oriented miRNAs do not account for the robust auxin-dependent primary root elongation observed in our transgenic lines. The specific identification of the miR396d interaction directly bridges this phenotypic gap. This indicates that OsAGO18 acts as a versatile pleiotropic hub, deploying distinct miRNA decoys—such as miR168/miR528 for antiviral immunity and miR396d for basal development—depending on the specific physiological context. This clearly suggests that the “sponge” or “decoy” activity of OsAGO18 is not an exclusive mechanism for immunity but a versatile post-transcriptional strategy utilized by monocots to fine-tune essential growth pathways.

The sequestration of miR396d by OsAGO18 functionally links this AGO protein to the well-established Growth-Regulating Factor (GRF) module. The miR396-*GRF* module is a master regulatory hub dictating plant architecture, cell proliferation, and phytohormone signaling [10,11]. Our qRT-PCR results confirmed that the expression of *OsGRF6*, a primary target of miR396d, is drastically de-repressed in *AGO18-OE* lines. Previous studies have established that elevated *OsGRF6* expression promotes auxin biosynthesis and signaling, leading to enhanced organ growth [11]. This molecular cascade perfectly aligns with our physiological observations: overexpression of *OsAGO18* significantly promoted IAA accumulation and subsequently drove massive primary root elongation. Conversely, the delayed seedling growth in *ago18* mutants further substantiates the necessity of the OsAGO18-miR396d-*OsGRF6* regulatory axis in maintaining robust early development. It is noteworthy that we observed an allele-specific phenotypic discrepancy between our two loss-of-function mutants. While both mutants exhibited drastically shorter primary roots compared to the massive elongation of the overexpression lines, the CRISPR-generated *C-ago18* mutant showed a primary root slightly longer than the wild-type on Days 4 and 5, whereas the T-DNA insertion mutant *ago18-T17* remained comparable to the wild-type (Figure 4C). This distinct pattern is likely attributable to genetic compensation—a well-documented phenomenon where deleterious frameshift mutations (such as the CRISPR/Cas9 indels in *C-ago18*) actively trigger the compensatory upregulation of homologous genes to maintain basal fitness, an effect often absent in large T-DNA insertion alleles. Given the highly redundant rice AGO family (e.g., the established functional overlap between OsAGO18 and AGO1 clade members such as OsAGO1d) [9,20], it is highly plausible that the mild growth “overshoot” in *C-ago18* is a side-effect of this broad compensatory re-adjustment. Crucially, unlike the *AGO18-OE* lines, neither mutant exhibited a significant surge in endogenous IAA levels (Figure 4D). This indicates that the massive root elongation in the OE lines is a specific consequence of the hyper-activated OsAGO18-miR396d-*OsGRF6*-auxin cascade, whereas the mutant phenotypes reflect the complex buffering capacity of the basal small RNA regulatory network.

Despite these compelling genetic, physiological, and in silico observations, we acknowledge a limitation in our current mechanistic model. While the re-analysis of public RIP-seq datasets and AlphaFold3 structural predictions strongly support the decoy hypothesis, direct in vivo biochemical evidence demonstrating the physical interaction between the OsAGO18 protein and the miR396d transcript is currently lacking in our specific experimental system. Future investigations employing targeted RNA immunoprecipitation coupled with qPCR (RIP-qPCR) assays at specific seedling developmental stages are necessary to definitively validate this physical sequestration. Nevertheless, the robust phenotypic outputs and downstream target validations presented here provide a highly plausible framework for this miRNA-decoy regulatory module.

### 3.3. Dual Anchorage: OsAGO18 Coordinates Development at the Expense of Fungal Resistance

The analysis of 34 transcriptome datasets from *M. oryzae* (Guy11) infection provides profound insights into the antagonistic role of *OsAGO18* in fungal immunity. Our WGCNA results show that the *OsAGO18*-associated network (MEyellow module) is highly active in healthy tissues but is sharply down-regulated upon fungal invasion across both resistant and sensitive genotypes (Supplementary Figure S1). In the context of plant pathology, this rapid suppression is highly strategic. Since our physiological data demonstrate that *OsAGO18* promotes growth via IAA accumulation, and given that auxin signaling is known to loosen cell walls and antagonize salicylic acid-mediated defense [21,22], *OsAGO18* likely functions as a negative regulator of rice blast resistance.

The anchorage of OsAGO18 to the growth machinery inevitably compromises its role in fungal defense, illustrating a classic “growth-defense tradeoff” [23,24]. Upon *M. oryzae* perception, the plant actively dismantles the OsAGO18-auxin axis to reinforce the cell wall and reallocate energy toward immunity. The stark contrast between its robust induction by viruses [8] and its rapid repression by fungi underscores the high functional plasticity of OsAGO18. Collectively, our study elucidates that OsAGO18 is a pivotal hub anchored to both seedling development and fungal susceptibility. This dual-anchorage highlights OsAGO18 as a prime target for molecular breeding to decouple growth from susceptibility, offering a strategy to optimize rice architecture without compromising broad-spectrum resilience.

## 4. Materials and Methods

### 4.1. Plant Materials and Growth Conditions

The wild-type cultivated rice used in this study was *O. sativa* subsp. *japonica* cv. ‘NPB’. NPB served as the uniform genetic background for all transgenic materials, including the *OsAGO18* overexpression lines (*AGO18-OE*) and the *ago18* mutants (*ago18-T17* and *C-ago18*). All materials were cultivated in a greenhouse in Nanjing, China, under controlled conditions of 28 °C temperature, 75% humidity, and a 12 h light/12 h dark cycle. Rice seeds were soaked in ultrapure water and incubated at 28 °C to induce germination, with daily water changes. The planting substrate consisted of a black soil-to-vermiculite mixture at a 3:1 ratio.

### 4.2. Isolation of Rice Protoplasts

Etiolated or normal green rice seedlings were cultured for 7–14 days. Upon reaching approximately 10 cm in height, the seedlings were uprooted and washed thoroughly with distilled water. For protoplast isolation, rice stem bases were cut into 0.5–1 mm strips using a razor blade. The sliced stem bases were immediately transferred to and fully submerged in an enzyme solution. Digestion was carried out in the dark for at least 3 h with gentle shaking (40 rpm/min) until the majority of the protoplasts were released from the stem base tissues. The digestion mixture was then transferred to a 50 mL centrifuge tube, and the protoplasts were pelleted by centrifugation at 100× *g* for 3 min (with both acceleration and deceleration set to 0). To prevent cell rupture, the resulting pellet was gently washed twice with 10 mL of ice-cold W5 solution (154 mM NaCl, 125 mM CaCl_2_, 5 mM KCl, and 2 mM MES, pH adjusted to 5.7 with NaOH). Finally, the washed protoplasts were resuspended in an appropriate volume of W5 solution and incubated on ice for at least 30 min. During this incubation period, the protoplast concentration was determined using a hemocytometer.

### 4.3. Protoplast Transformation

For plasmid transformation, 10 μL (10–20 μg) of plasmid DNA and 100–150 μL of the prepared protoplast suspension were added to a sterile 2 mL microcentrifuge tube and mixed gently. An equal volume of PEG 4000 solution (40% (*w*/*v*) PEG 4000, 0.6 M mannitol, and 100 mM CaCl_2_) was subsequently added to the mixture, followed by gentle homogenization. The transfection mixture was incubated at 28 °C for 20 min. The reaction was then terminated by adding 500 μL of W5 solution, and the protoplasts were pelleted by centrifugation at 450× *g* for 3 min. The PEG-containing supernatant was carefully discarded, and the resulting pellet was resuspended in 750 μL of W5 solution. The transformed protoplasts were incubated in the dark at 28 °C for 12–16 h. Following incubation, fluorescence signals were observed using a confocal laser scanning microscope (LSM, Carl Zeiss, Oberkochen, Germany).

### 4.4. Acquisition and Processing of Public RNA-Seq Datasets

A total of 34 publicly available high-resolution transcriptome datasets were retrieved from the NCBI Sequence Read Archive (SRA) database and utilized in this study. As detailed in Supplementary Table S1, these datasets capture the transcriptomic dynamics of diverse rice genotypes—including the wild-type NPB, the highly susceptible line LTH, and the broad-spectrum resistant line IRBLkm-Ts—following inoculation with the blast fungus *M. oryzae* (strain Guy11) across multiple early time points. The raw paired-end sequencing data were subjected to strict quality control. FastQC (v0.11.9) was utilized to evaluate the sequence quality of individual samples, and the results were aggregated into a global quality report using MultiQC (v1.14). To obtain high-quality clean reads, adapter sequences and low-quality bases were removed using Trim Galore (v0.6.7), which incorporates the cutadapt tool. To eliminate ribosomal RNA (rRNA) contamination, the trimmed reads were aligned to the rice rRNA reference database using Bowtie2 (v2.4.4), and the mapped reads were discarded.

The resulting purified clean reads were mapped to the *O. sativa* reference genome using HISAT2 (v2.2.1). SAMtools (v1.13) was then used to convert the alignment output into BAM format and sort the files. For gene expression quantification, featureCounts (v2.0.3) was employed to calculate the number of reads mapped to each gene, generating a raw gene expression count matrix. Based on this matrix, data normalization and subsequent analyses were conducted using the DESeq2 package (v1.38.0) in R (v4.2.1). The normalized dataset was ultimately utilized to screen for co-expressed genes significantly correlated with the *OsAGO18* expression pattern.

Based on the normalized expression matrix, two distinct downstream analytical approaches were performed. For single-gene enrichment analysis, Pearson correlation coefficients between the expression level of *OsAGO18* and all other genes were calculated, and the top-ranked correlated genes were selected for GO and KEGG enrichment analyses. Concurrently, the exact same normalized matrix was subjected to WGCNA to construct a global co-expression network and partition genes into specific modules.

### 4.5. Plasmid Construction and Genetic Transformation

To generate the OsAGO18 overexpression (AGO18-OE) lines, the full-length coding sequence of OsAGO18 (100% identical to the reference sequence) was amplified directly from the cDNA of the NPB cultivar using specific primers (AGO18-Clone-F and AGO18-Clone-R primers). The resulting amplicon was then cloned into the pCAMBIA1300-35S-Flag binary vector via standard restriction enzyme digestion and ligation. The constructed vectors were introduced into *Agrobacterium tumefaciens* (strain EHA105) and subsequently transformed into rice calli following standard *Agrobacterium*-mediated transformation protocols. Among the generated independent transgenic lines, *AGO18-OE* lines No. 15 and 20 were selected for all subsequent phenotypic and molecular analyses because they exhibited the highest and most stable *OsAGO18* overexpression levels (Supplementary Figure S2), thereby ensuring phenotypic reproducibility and minimizing potential genomic position effects. The *OsAGO18* insertion mutant line was obtained from the rice *Tos17* mutant database (https://tos.nias.affrc.go.jp/). Additionally, the *C-ago18* mutant was generated using the CRISPR/Cas9 system. Specific single guide RNA (sgRNA) target sequences were designed via the online web tool (http://crispr.hzau.edu.cn/) and inserted into the pYLCRISPR/Cas9Pubi vector according to previously established protocols [25].

The coding sequence of *OsAGO18* was cloned into the pYL322d1-eGFP-C vector via homologous recombination. The specific primers used for vector construction are listed in Supplementary Table S2.

### 4.6. Quantitative RT-PCR

Total RNA was extracted from rice samples using TRIzol reagent (Invitrogen, Carlsbad, CA, USA). First-strand cDNA was synthesized from 1 μg of total RNA utilizing the PrimeScript RT Reagent Kit (Takara, Kusatsu, Japan). QRT-PCR was performed using the 2× AceQ qPCR SYBR Green Master Mix (Vazyme, Nanjing, China) on an ABI 7500 Real-Time PCR System (Thermo Fisher Scientific, Waltham, MA, USA). Primer sequences used in this study are provided in Supplementary Table S2.

### 4.7. Statistical Analysis

All experiments in this study were performed with at least three independent biological replicates. Quantitative data are presented as the mean ± standard deviation (SD). Statistical significance between the control and experimental groups was determined using the two-tailed Student’s *t*-test. The differences were considered statistically significant at specific thresholds, indicated by asterisks in the figures (* *p* < 0.05, ** *p* < 0.01, and *** *p* < 0.001). Initial data processing and basic calculations were performed using Microsoft Excel. Subsequent formal statistical analyses and data visualization were conducted using GraphPad Prism 9.0 software.

## 5. Conclusions

In conclusion, our study reveals a novel, pleiotropic role for the monocot-specific OsAGO18 in coordinating basal plant development. Moving beyond its well-established function in antiviral immunity, we demonstrate that OsAGO18 actively promotes early seedling growth and primary root elongation under normal physiological conditions. Mechanistically, our integrated analysis suggests that OsAGO18 localizes to P-bodies and functions as a putative molecular decoy for miR396d. This proposed sequestration likely relieves the post-transcriptional repression of *OsGRF6*, thereby activating downstream auxin-dependent developmental cascades. Furthermore, the rapid transcriptomic suppression of this growth-related network upon fungal infection highlights the functional plasticity of OsAGO18 in navigating the growth-defense tradeoff. Ultimately, these findings provide a comprehensive framework for understanding how a single AGO protein integrates small RNA regulation, phytohormone homeostasis, and environmental responsiveness, offering potential molecular targets for breeding resilient rice varieties.

## Data Availability

The data that support the findings of this study are presented in the Supplementary Table S1.

## Supplementary Materials

The following supporting information can be downloaded at: https://www.mdpi.com/article/10.3390/plants15101580/s1, Figure S1: Weighted Gene Co-expression Network Analysis of the transcriptome datasets. The heatmap displays the module-trait relationships to identify gene networks associated with diverse physiological states. Each row represents a specific co-expression module (labeled by distinct colors on the left), and each column corresponds to a specific condition or developmental baseline (time0 to time72, resistant, sensitive, inoculated, normal). Each cell contains the Pearson correlation coefficient between the module and the trait, with the corresponding *p*-value displayed in parentheses. The color gradient from blue to red represents the correlation scale from −1 (strong negative correlation) to 1 (strong positive correlation). *OsAGO18* is assigned to the MEyellow module, which exhibits a highly significant positive correlation specifically with the “normal” developmental baseline and negative correlations with stress-induced states; Figure S2: Identification of transgenic plants. (A) QRT-PCR analysis of *OsAGO18* expression in the wild-type control, *OsAGO18* overexpression lines, and the *ago18-T17* mutant. (B) Sanger sequencing confirmation of the targeted mutation in the *C-ago18* mutant line, revealing a 7-base pair deletion at positions 1197–1203 bp within the genomic coding sequence. For qRT-PCR, *18s-rRNA* was used as the internal reference for normalization. Values are represented as mean ± SD. Asterisks indicate significant differences compared to the NPB control according to Student’s *t*-test (*** *p* < 0.001); Figure S3: Expression analysis of IAA-related genes in *OsAGO18* transgenic lines. QRT-PCR analysis of the auxin signaling gene *OsIAA10* (A) and the auxin efflux carrier gene *OsPIN9* (B) in the wild-type (NPB), *OsAGO18* overexpression lines, and *ago18* mutant lines. The relative expression levels were normalized to the internal control. *18S rRNA* was used as the internal reference for normalization. Values are represented as mean ± SD from three independent biological replicates. Asterisks indicate significant differences compared to the NPB control according to Student’s *t*-test (** *p* < 0.01, *** *p* < 0.001); Table S1: Summary of RNA-seq data and read mapping statistics; Table S2: The primers used in this study.
