# Supplementary material for: Overexpression of OsAGO18 Promotes Early Seedling Development and Root Elongation in Rice
Source: Plants (Basel). 2026 May 21;15(10):1580. doi: 10.3390/plants15101580 (PMC13210671; doi:10.3390/plants15101580)
Supplement: Supplementary file 1 [file plants-15-01580-s001.zip › Supplementary Figures.pdf]

Supplemental Figures for

OsAGO18 **Promotes Early Seedling Development and Coordinates a Growth-Related Transcriptional Network Responsive to Fungal Infection in Rice**

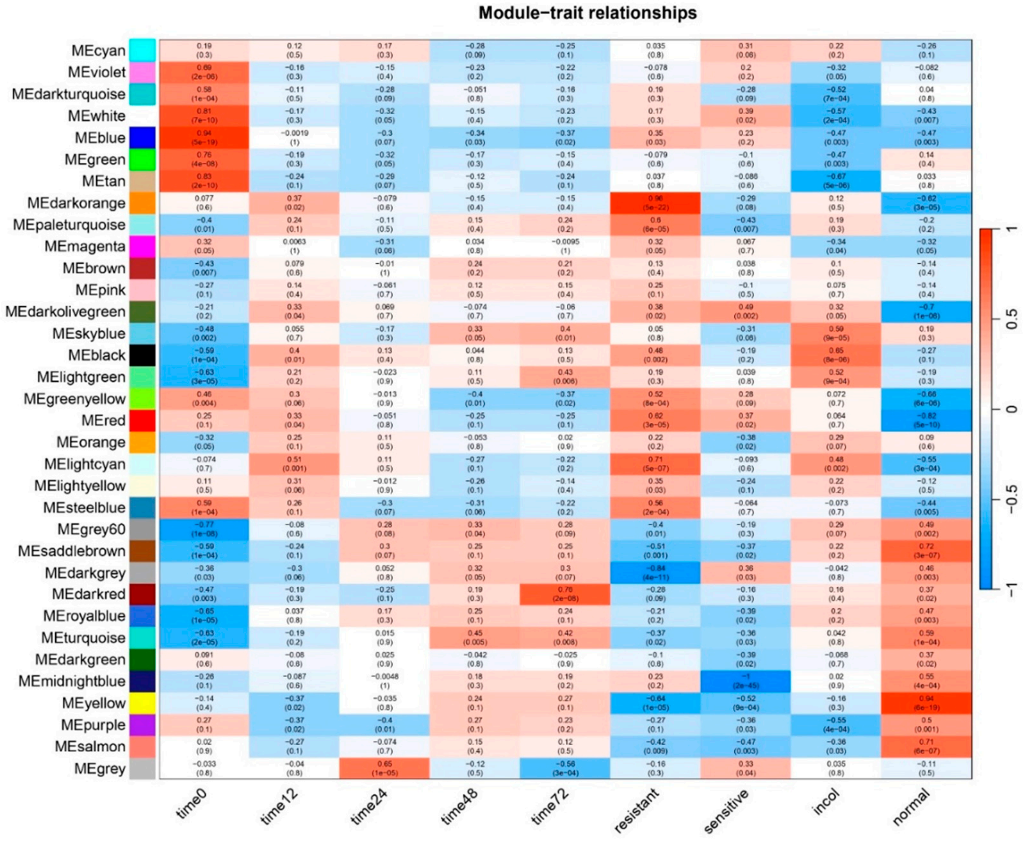

Supplementary Figure S1. Weighted Gene Co-expression Network Analysis of the transcriptome datasets. The heatmap displays the module-trait relationships to identify gene networks associated with diverse physiological states. Each row represents a specific co-expression module (labeled by distinct colors on the left), and each column corresponds to a specific condition or developmental baseline (time0 to time72, resistant, sensitive, inoculated, normal). Each cell contains the Pearson correlation coefficient between the module and the trait, with the corresponding p-value displayed in parentheses. The color gradient from blue to red represents the correlation scale from -1 (strong negative correlation) to 1 (strong positive correlation). *OsAGO18* is assigned to the MEyellow module, which exhibits a highly significant positive correlation specifically with the "normal" developmental baseline and negative correlations with stress-induced states.

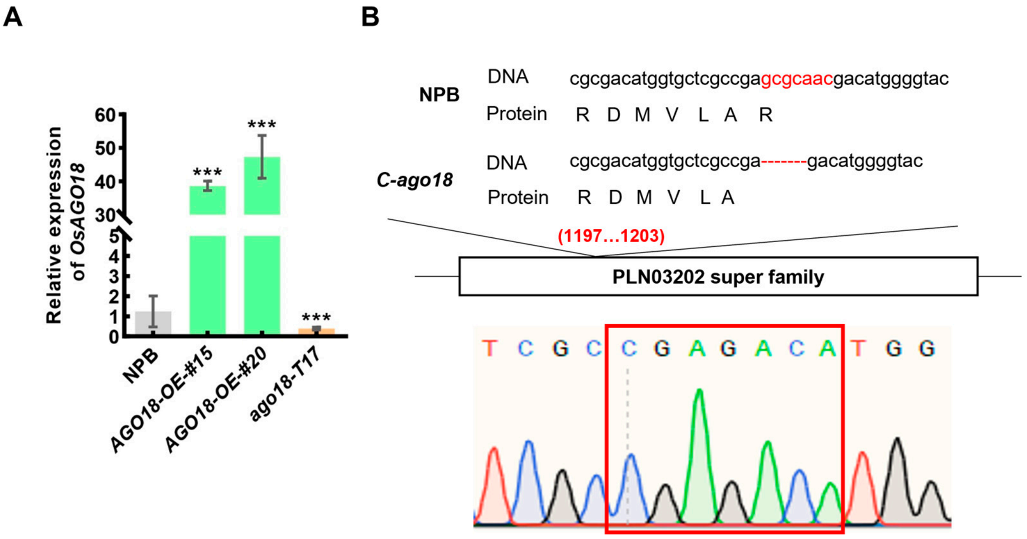

Supplementary Figure S2. Identification of transgenic plants. (A) QRT-PCR analysis of *OsAGO18* expression in the wild-type control, *OsAGO18* overexpression lines, and the *ago18-T17* mutant. (B) Sanger sequencing confirmation of the targeted mutation in the *C-ago18* mutant line, revealing a 7-base pair deletion at positions 1197–1203 bp within the genomic coding sequence. For qRT-PCR, *18s-rRNA* was used as the internal reference for normalization. Values are represented as mean ± SD. Asterisks indicate significant differences compared to the NPB control according to Student's t-test (*** $p < 0.001$).

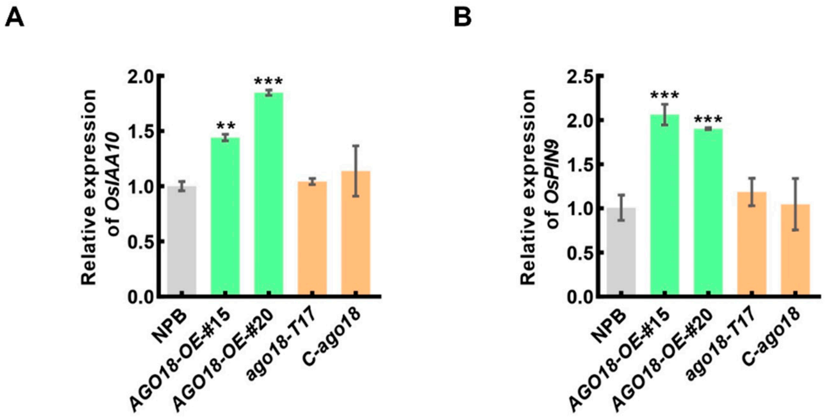

Supplementary Figure S3. Expression analysis of IAA-related genes in *OsAGO18* transgenic lines. QRT-PCR analysis of the auxin signaling gene *OsIAA10* (A) and the auxin efflux carrier gene *OsPIN9* (B) in the wild-type (NPB), *OsAGO18* overexpression lines, and *ago18* mutant lines. The relative expression levels were normalized to the internal control. *18S rRNA* was used as the internal reference for normalization. Values are represented as mean ± SD from three independent biological replicates. Asterisks indicate significant differences compared to the NPB control according to Student's t-test (** $p < 0.01$, *** $p < 0.001$).
